# Costs of Digital Adherence Technologies for Tuberculosis Treatment Support, 2018–2021

**DOI:** 10.3201/eid3001.230427

**Published:** 2024-01

**Authors:** Ntwali Placide Nsengiyumva, Amera Khan, Maricelle Ma. Tarcela S. Gler, Mariecef L. Tonquin, Danaida Marcelo, Mark C. Andrews, Karine Duverger, Shahriar Ahmed, Tasmia Ibrahim, Sayera Banu, Sonia Sultana, Mona Lisa Morales, Andre Villanueva, Egwumo Efo, Baraka Onjare, Cristina Celan, Kevin Schwartzman

**Affiliations:** McGill International Tuberculosis Centre and Research Institute, McGill University Health Centre, Montreal, Quebec, Canada (N.P. Nsengiyumva, K. Schwartzman);; Stop TB Partnership, Geneva, Switzerland (A. Khan);; De La Salle Medical and Health Sciences Institute, Makati City, Philippines (M.M.T.S. Gler, M.L. Tonquin, D. Marcelo);; Health Through Walls, Port au Prince, Haiti (M.C. Andrews, K. Duverger);; icddr,b, Dhaka, Bangladesh (S. Ahmed, T. Ibrahim, S. Banu, S. Sultana);; KNCV, Manila, Philippines (M.L. Morales, A. Villanueva);; KNCV, Dar es Salaam, Tanzania (E. Efo, B. Onjare);; Center for Health Policies and Studies, Chisinau, Moldova (C. Celan)

**Keywords:** Tuberculosis, tuberculosis and other mycobacteria, digital adherence technologies, cost, tuberculosis, pill sleeves, video-observed treatment, Bangladesh, Haiti, Moldova, the Philippines, Tanzania, bacteria

## Abstract

Digital adherence technologies are increasingly used to support tuberculosis (TB) treatment adherence. Using microcosting, we estimated healthcare system costs (in 2022 US dollars) of 2 digital adherence technologies, 99DOTS medication sleeves and video-observed therapy (VOT), implemented in demonstration projects during 2018–2021. We also obtained cost estimates for standard directly observed therapy (DOT). Estimated per-person costs of 99DOTS for drug-sensitive TB were $98 in Bangladesh (n = 719), $119 in the Philippines (n = 396), and $174 in Tanzania (n = 976). Estimated per-person costs of VOT were $1,154 in Haiti (87 drug-sensitive), $304 in Moldova (173 drug-sensitive), $452 in Moldova (135 drug-resistant), and $661 in the Philippines (110 drug-resistant). 99DOTS costs may be similar to or less expensive than standard DOT. VOT is more expensive, although in some settings, labor cost offsets or economies of scale may yield savings. 99DOTS and VOT may yield savings to local programs if donors cover infrastructure costs.

As part of the mission to cure and ultimately eliminate tuberculosis (TB), maintaining treatment adherence poses a substantial barrier (*1*). Persons with TB must complete multidrug regimens typically lasting >6 months. Even small lapses in adherence can be associated with poorer treatment outcomes, including relapse with the potential for further transmission (*2*). TB prevention and care programs have often sought to improve adherence, and hence treatment outcomes, by using directly observed therapy (DOT) (*3*,*4*). However, healthcare system barriers (mostly resource limitations), coupled with stigma, loss of autonomy, and the heavy burden of DOT clinic visits, can result in subpar outcomes and adherence that may not exceed that of self-administered treatment (*5*–*8*). Those limitations have led the World Health Organization (WHO) to recommend community or home-based DOT over healthcare facility–based DOT or unsupervised treatment (*4*).

WHO defines a DOT provider as any person who observes the person with TB taking their medications in real time (*4*). By leveraging current advances in mobile technologies, person-centered treatment observation can be achieved by digital adherence technologies (DATs) such as medication sleeves, smart pill boxes, and video-supported therapy. Moreover, real-time digital adherence information offers the possibility of tailoring treatment support to individual needs. However, before TB programs adopt those technologies as a central strategy for treatment support, evidence for their effectiveness must be robust. Demonstration projects highlighting feasibility and acceptability of DATs for TB treatment support provide substantial evidence; to date, evidence is more limited for clinical outcomes with use of DATs than for other forms of treatment observation or self-administered treatment (*9*–*11*). In principle, DATs can enable expansion of TB treatment supervision and support while reducing the burden on persons with TB and their providers.

Information about the cost to TB programs of those technologies and their real-world cost-effectiveness comes largely from pilot and modeling studies (*11*,*12*). To estimate the cost of 2 DATs currently recommended for use by WHO (*4*), we used data from implementation studies in Bangladesh, Haiti, Moldova, the Philippines, and Tanzania. Each demonstration project participating in this study received local institutional ethics review board approval.

## Methods 

### Study Design and Tools

Our multicountry cost analysis reflects DAT implementation projects funded by TB REACH Wave 6 (*13*). Our goal was to estimate capital and recurrent costs of DATs. We developed questionnaires and measurement tools to describe the current standard of care (without DATs) for treatment of drug-susceptible TB (DS-TB) and drug-resistant TB (DR-TB); document how the DATs used in each project were integrated into the local standard of care and what practice changes resulted from their introduction; and capture all cost components of the DAT used during each project, including all related interventions and practice changes. Participating programs were given a series of cost analysis questionnaires, available online (*14*) and were trained in their use via webinars. Our report is limited to cost analyses and does not address cost-effectiveness. During execution of each project, we collected data prospectively.

### Study Populations

During 2018–2021, we enrolled eligible adolescents and adults receiving treatment for DS-TB and DR-TB in DAT implementation projects in Bangladesh, Haiti, Moldova, the Philippines, and Tanzania (Table 1). The projects were either 99DOTS (a technology-enabled supplement to enhanced DOT (https://www.99dots.org/About.html) medication sleeves or video-observed therapy (VOT; also referred to as video-supported therapy).

**Table 1:** Implementation projects and participants in study of costs of digital adherence technologies for tuberculosis treatment support*

Country	DAT	Organization	Project description	Participants
Bangladesh	99DOTS†	icddr,b	Implementation of 99DOTS in private sector TB screening and treatment centers established by icddr,b under its social enterprise model in Dhaka	719 adults with DS-TB (mean age 34 y, SD 27) enrolled in the project, April 2019–July 2020.
Philippines	99DOTS	KNCV	A project to assess 99DOTS use in the private sector in the Philippines, where data suggest that 50% of patients in the country seek care	396 adults (>15 y) with DS-TB enrolled on 99DOTS at 3 private clinics based in metro Manila area, December 2018–June 2020.
Tanzania	99DOTS	KNCV	The project involved mining communities in Tanzania in 4 rural districts using 99DOTS with SMS-targeted educational messages and reminders. Patient dosing histories were used for counseling and for differentiated care (more intensive patient management)	976 adult miners (>15 y) with DS-TB from 11 public health facilities recruited at treatment initiation or during medication refill. 22 DOT nurses and 11 community health workers were engaged in the project, February 2019–June 2020.
Haiti	VOT	Health through Walls, Inc.	A project using VOT to improve TB treatment adherence and outcomes for current and former prisoners in Haiti	87 incarcerated persons with DS-TB enrolled in the project, February 2019–February 2020. The project used a commercial VOT platform.
The Philippines	VOT	Hermano (San) Miguel Febres Cordero Medical Education Foundation, Inc	A pilot study to determine feasibility and acceptability of VOT in a high-burden, resource constrained DR-TB clinic in the Philippines, where smartphone penetration is moderate and growing	110 adolescents and adults (>13 y) with DR-TB enrolled to use VOT at 6 DR-TB clinics. Everyone received a smartphone with the VOT application pre-installed and with SIM cards, December 2018–June 2021. The project used a commercial VOT platform.
Moldova	VOT	Centre for Health Policies and Studies	A pilot project to scale up a locally developed VOT application	173 adults with DS-TB and 135 adults with DR-TB enrolled on a locally developed VOT platform, April 2020–June 2021.

*Numbers indicate participants in the costing exercise; the overall parent study might have had more participants. CHW, community health worker; DAT, digital adherence technology; DR-TB, drug-resistant TB; DS-TB, drug-susceptible TB; KNCV, Koninklijke Nederlandse Centrale Vereniging tot bestrijding der Tuberculose; TB, tuberculosis; VOT, video-observed therapy; VST, video-supported therapy; SMS,short message service. †https://www.99dots.org.

### Cost Analyses

We performed a combination of bottom-up and top-down microcosting for 6 TB REACH (https://tbreach.org) DAT implementation projects. Seven other implementation projects pursued separate costing analyses, some of which have been published elsewhere (e.g., the Ugandan project [*15*]). Bottom-up costing applied to most cost components, but top-down microcosting incorporated total amounts spent for DAT platform/infrastructure, systems/data management and technical support, and training activities. Bottom-up microcosting involves a detailed enumeration of cost component data points obtained from the service provider to estimate unit costs (*16*,*17*). Top-down microcosting uses total costs for relevant elements and then prorates them to calculate unit costs (e.g., per patient treated, per service delivered) (*18*,*19*).

We conducted our analysis from the healthcare system perspective; hence, we did not tabulate costs borne by patients and their families. All costs were converted to 2022 US dollars by using the respective countries’ inflation and exchange rates from the World Bank (*20*,*21*). Costs reflected project expenses reported by staff and were grouped into 2 categories: fixed and variable. Variable costs fell into 7 categories: 1) phones and accessories; 2) systems/data management; 3) mobile data use; 4) adherence monitoring by healthcare workers (HCWs) using the DAT platform; 5) HCW escalation/intervention in cases of nonadherence; 6) trainers; and 7) trainees. Two additional variable cost categories were specific to 99DOTS: 1) printing and shipping of medication sleeves, and 2) medication preparation (in case medication blister packs were not packaged within 99DOTS sleeves by the supplier). Fixed costs were those of the DAT platform and required infrastructure (Appendix).

For each project, we estimated total costs and then prorated them per person treated for TB. Because certain fixed costs (e.g., acquisition of the relevant platforms and computing infrastructure) can be substantial, we performed scenario analyses in which the DAT was scaled up to support more persons during treatment. In those scenarios, most variable costs remained unchanged, but we annuitized fixed technology costs as well as computer and phone purchases for HCWs and patients on the basis of a 5-year lifespan. We also explored scenarios in which fixed technology/platform introduction and maintenance costs would be shared across expanded user numbers (i.e., 2× study population, 5× study population, 10× study population, and 100× study population) while maintaining the same variable costs as previously estimated.

To situate the DAT costs relative to the local standard of care; we evaluated per-person costs for DATs compared with in-person DOT, accounting for each study setting (i.e., duration of treatment and salary/wages of the persons observing treatment). We supplemented this analysis by considering DOT costs from the existing literature. To capture the patient volumes at which DAT scale-up might become cost saving, we also performed a threshold analysis.

An additional scenario analysis accounted for the high fixed up-front costs, which can pose a substantial barrier to DAT adoption. In that scenario analysis, we assumed that the costs were covered separately by international donor funds. Thus, we excluded them from the analysis, which was therefore restricted to variable costs borne by the local TB program, including computer and phone purchases for HCWs and patients. We then compared those estimates with the cost estimates for DOT.

## Results 

During 2018–2021, the three 99DOTS projects enrolled a total of 2,091 patients: 719 in Bangladesh, 396 in the Philippines and 976 in Tanzania. During the same period, the 3 VOT projects enrolled a total of 505 patients: 87 in Haiti, 308 in Moldova, and 110 in the Philippines (Tables 1, 2; Appendix Tables 2–4, 6–9). 

**Table 2:** Costs of digital adherence technologies for tuberculosis treatment support determined in study of costs of digital adherence technologies for tuberculosis treatment support*

**Adherence technology**	**Variable costs, US$**			**Fixed costs, US$**			**Total costs, US$**	
	Variable overall	Variable per patient		Fixed overall	Fixed per patient		Total overall	Total per patient
Observed project costs								
99DOTS†								
Bangladesh, n = 71	55,985	78		14,772	21		70,756	98
The Philippines, n = 396	33,173	84		13,901	35		47,074	119
Tanzania, n = 976	159,028	163		10,509	11		169,536	174
VOT								
Haiti, n = 87	69,287	796		31,149	358		100,436	1,154
Moldova, DS-TB, n = 173	34,248	198		18,429	107		52,677	304
Moldova DR-TB, n = 135	40,088	293		21,571	160		61,659	452
Moldova all TB, n = 308	74,336	242		40,000	130		114,336	372
The Philippines, n = 110	45,330	412		27,327	248		72,656	661
Project costs annuitized over the 5-y life span of servers and phones								
99DOTS								
Bangladesh, n = 719		60			21			81
The Philippines, n = 396		83			8.84			92
Tanzania, n = 976		160			3.61			163
VOT								
Haiti, n = 87		702			358			1,060
Moldova DS-TB, n = 173		151			21			172
Moldova DR-TB, n = 135		222			32			254
Moldova All TB, n = 308		185			26			211
The Philippines, n = 110		247			248			495

*DR-TB, drug-resistant TB; DS-TB, drug-susceptible TB; TB, tuberculosis; VOT, video-observed treatment. †https://www.99dots.org.

### 99DOTS

The estimated total costs of 99DOTS in the 3 implementation projects were $70,756 overall and $98 per person treated for DS-TB in Bangladesh, $47,074 overall and $119 per person in the Philippines, and $169,536 overall and $174 per person in Tanzania. Variable costs accounted for 79% of the total in Bangladesh, 70% in the Philippines, and 94% in Tanzania (Appendix Tables 3, 5, 6).

The main cost drivers for 99DOTS varied across project sites. Key components included adherence monitoring by personnel in Bangladesh (25% of costs), platform and infrastructure in the Philippines (29%), and systems/data management in Tanzania (34%).

**Figure 1:**
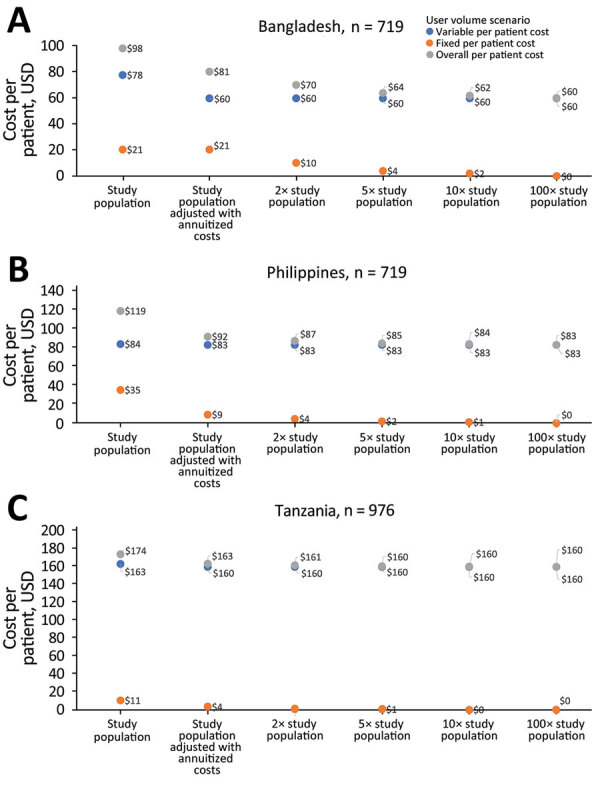
Directly observed tuberculosis therapy scale-up scenario analysis for 3 countries: A) Bangladesh; B) the Philippines; C) Tanzania. In each scenario, fixed technology/platform introduction and maintenance costs are shared across expanded user numbers (i.e., 2× study population, 5× study population, 10× study population, and 100× study population) while maintaining the same variable costs.

For scenario analyses, we evaluated variation in per-person costs if 99DOTS were potentially scaled to larger numbers of persons receiving TB treatment and of their providers, while maintaining the same total fixed costs and the same variable costs per patient. When the number of persons served by the platform was increased 100-fold, we estimated a 39% decrease in cost per patient to $60 in Bangladesh, a 30% decrease to $83 in the Philippines, and an 8% decrease to $160 in Tanzania (Figure 1). In all scenario analyses, the most influential cost components remained the same (they all belonged to the variable cost category). 

### VOT

For the project in Haiti (DS-TB), estimated overall costs for VOT were $100,436 and costs per person treated were $1,154; for Moldova (all TB patients), $114,336 and $372; and for the Philippines (DR-TB), $72,656 and $661. Variable costs accounted for 69% of the total in Haiti (DS-TB), 65% in Moldova (all TB patients), and 62%, in the Philippines (DR-TB) (Appendix Tables 6–8).

The largest cost component in Haiti was associated with systems/data management and technical support, which together accounted for 54% of the total. VOT platform and infrastructure accounted for 35% of the total cost in Moldova and 38% in the Philippines.

**Figure 2:**
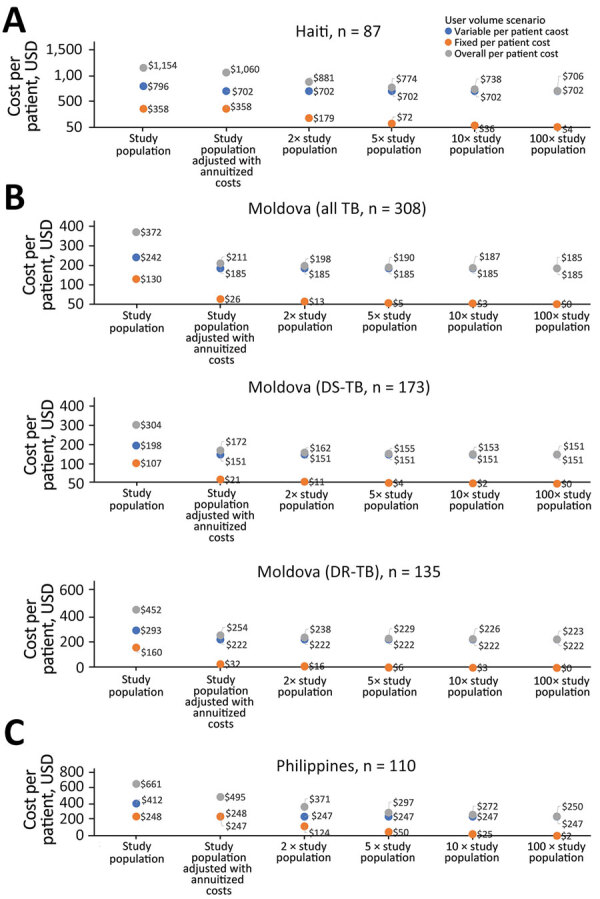
Video-observed tuberculosis therapy scale-up scenario analysis for 3 countries: A) Haiti; B) Moldova; C) the Philippines. In each scenario, fixed technology/platform introduction and maintenance costs are shared across expanded user numbers (i.e., 2× study population, 5× study population, 10× study population, and 100× study population) while maintaining the same variable costs. All TB, all TB patients; DR-TB, drug-resistant TB; DS-TB, drug-susceptible TB; TB, tuberculosis.

For the scenario analysis, when the number of persons served by the same platform was increased 100-fold, we estimated a 31% decrease in cost with per-person costs falling to $800 in Haiti (DS-TB), 12% falling to $185 in Moldova (all TB patients), and 50% falling to $151 in the Philippines (DR-TB) (Figure 2). In that scenario, the largest cost component in Haiti (DS-TB) remained systems/data management and technical support at 91%; adherence monitoring then accounted for 55% of the total per patient cost in Moldova (all TB patients) and 88% in the Philippines. 

### 99DOTS Versus DOT

The 99DOTS projects were conducted in settings in which TB treatment is ordinarily observed by health facility workers, community health workers, or family members. Program personnel estimated that 30% of patients would continue traditional DOT after the introduction of 99DOTS in Bangladesh, 20% in the Philippines, and 0 in Tanzania. Such persons included those receiving TB retreatment, persons without access to a mobile phone, persons residing outside the clinic’s catchment area, hospitalized patients, persons with extrapulmonary TB receiving >12-month treatment regimens, and persons unwilling to provide consent. From the Tanzania healthcare system perspective, DOT itself does not imply healthcare system costs, and hence DOT costs to the healthcare system are not offset by use of 99DOTS because treatment support is ordinarily provided (unpaid) by family members.

In a threshold analysis, we explored the patient volumes required for 99DOTS to be cost saving when compared with DOT. In Bangladesh, when we used study DOT cost estimates, 99DOTS was associated with a $7 increase per patient enrolled; when we used DOT costs published elsewhere, the incremental cost was $45 (*22*). We estimated that an increase of >47% in patient volumes from the study population would render 99DOTS cost saving. In the Philippines, 99DOTS was cost saving with existing patient volumes according to study DOT cost estimates and those published elsewhere (*23*). In Tanzania, there was no possibility of healthcare system cost savings with 99DOTS compared with DOT because DOT relied on family members at no cost to the healthcare system.

For the scenario in which fixed costs are paid by donors, 99DOTS was cost saving in Bangladesh and the Philippines. In that scenario, TB programs would be able to support treatment for $60 per patient by using 99DOTS compared with $74 per patient by using DOT in Bangladesh, $83 compared with $176 in the Philippines, and $160 compared with $0 per patient in Tanzania.

### VOT versus DOT

Program personnel estimated that 15% of persons treated for TB would remain on traditional DOT after the implementation of VOT in Haiti, 10% in Moldova, and 50% in the Philippines (Table 3). Use of VOT in Haiti was associated with $646 additional cost per patient compared with study DOT cost estimates and $1,011 compared with published DOT cost estimates (*25*). In Moldova, VOT was cost-saving with existing patient volumes according to our own DOT cost estimates; 77% of the DS-TB and 75% of the DR-TB study populations actually enrolled would have been sufficient to generate cost savings in Moldova. In Bangladesh and the Philippines, VOT costs per patient exceeded those for DOT even with all fixed costs excluded, meaning that expanding the number of patients covered by VOT (i.e., economies of scale for fixed costs) could not result in savings.

**Table 3:** Digital adherence technologies compared with directly observed therapy costs*

Cost	99DOTS sites, US$				VOT sites, US$				
	Bangladesh, healthcare facility	Philippines, health care facility	Tanzania, home		Haiti, prison				Philippines, healthcare facility or home
						Moldova, health facility			
						DS-TB	DR-TB	All TB	
Crude estimate for DOT cost for the standard of care derived from the costing tool									
Hourly wage of person offering DOT support	0.83	1.40	0.00		4.60	11.20	11.20	11.20	2.88
Duration of DOT visit, min	45	45	0		45	15	15	15	2
Total per patient cost									
DOT cost from study data	74	176	0		414	336	504	427	17
DOT cost from the literature	36 (*22*)	155 (*23*)	89 (*24*)		49 (*25*)				166 (*23*)
Annuitized cost of DAT	81	92	163		1,060	172	254	211	495
Variable DAT costs only	60	83	160		702	151	222	185	247
Incremental per patient cost for the DAT versus standard of care									
Study data as comparator†	7	–84	163		646	–164	–250	–216	478
Prior published data as comparator‡	45	–63	74		1,011	–	–	–	329
Incremental per patient cost for the DAT using variable costs only versus standard of care, assuming fixed costs are already sunk									
Study data as comparator†	–14	–93	160		288	–185	–282	–242	230
Prior published data as comparator‡	24	–72	71		653	–	–	–	81

*DAT, digital adherence technologies; DOT, directly observed therapy; –, no prior published data available for comparison. †Negative incremental costs indicate savings for the DAT relative to standard of care. ‡There is no available literature documenting the cost of DOT in Moldova; therefore, we did not calculate incremental costs when published literature was the comparator.

For the scenario in which fixed costs are covered by donors, VOT was cost saving in Moldova but not in Haiti or the Philippines. In that scenario, TB programs would be able to support treatment with VOT for $702 per patient compared with $414 per patient with DOT in Haiti, $151 compared with $336 in Moldova (DS-TB), $222 compared with $504 in Moldova (DR-TB), $185 compared with $427 in Moldova (all TB patients), and $247 compared with $17 per patient in the Philippines.

## Discussion 

Our cost analysis for 2 DATS covered a wide range of settings with diverse populations, socioeconomic conditions, and TB epidemiology. Implementation costs, particularly infrastructure and training costs, were substantial when limited to a small number of persons with TB. However, if the DAT programs are scaled up to cover larger numbers of persons, the healthcare system cost per treatment course would fall and could become less expensive than paid in-person DOT.

The same infrastructure can be stretched only so far; a better knowledge of its capacity will be essential for understanding cost and budgetary effects of DAT expansion. Moving forward, it will also be useful to account for potential cost savings to the healthcare system, to the extent that in-person DOT is reduced and especially for potential cost savings to persons and families affected by TB.

Among the strengths of our analysis are a diversity of real-world settings, reflective of those in which TB care is provided, and the use of carefully gathered microcosting data. We explicitly considered scenarios in which user volume could be expanded to better harness the necessary technical infrastructure. However, the numbers of persons with TB included in these projects varied, and the project settings were not always representative of the available resources and infrastructure in some places where persons with TB obtain care. According to questionnaire responses from local project personnel, DOT visits were substantially longer in the Philippines and Bangladesh than at the other project sites. Nonetheless, those data provide relevant insights for managers and policy makers considering adding those technologies to their TB treatment programs.

We did not evaluate effectiveness of the DATs, which has been addressed in other related publications; an analysis of the feasibility and acceptability of DATs in the TB REACH projects is forthcoming (*26*). Limitations of the technologies themselves have been recognized: for example, messages received or not received by the 99DOTS platform do not necessarily equate with medication ingestion or lack thereof (*27*,*28*). Hence, direct comparisons of DAT and DOT costs are only appropriate to the extent that clinical outcomes with the DAT in question are similar to or better than standard care; we explicitly did not address that point in our analysis. Our study considered only economies of scale resulting from sharing of fixed infrastructure and equipment costs by more users. We did not have specific information as to how variable costs (e.g., HCW time, phones, and data charges) might fall with increased user numbers (e.g., greater HCW efficiency, discounted phones, and data with larger bulk purchasing). Clearly, any reductions in variable costs at scale would be relevant to providers and payers.

Of note, we did not evaluate costs or savings for patients and family members with regard to constraints such as missed work hours or childcare needs, which may be mitigated when in-person DOT is replaced by digital treatment support. Other studies have highlighted the value of such savings in the DAT context (*29*,*30*).

Among the limitations to our comparison of DATs with DOT, the total estimated cost for DOT was based on the reported staff time cost per DOT visit multiplied by the number of DOT visits expected for a complete course of treatment (perfect attendance). However, for 99DOTS and VOT, we included only the costs of the actual calls made and videos sent. On the other hand, we had explicit microcosting information, which enabled us to include the cost of escalation (additional phone calls and home visits) in the case of suboptimal adherence detected with the DATs. We did not have such granular data available for escalation costs in the event of suboptimal adherence during DOT. Similarly, we did not include DOT training costs because we did not have data for those.

The high costs of DATs, especially VOT, are driven largely by up-front infrastructure costs such as computing equipment and phones, initial configuration, and software licensing (Appendix Table 3). For Haiti and Moldova, where the cost of purchasing phones accounted for a substantial portion of the per patient cost, the loan of phones to patients or their use of personal phones would drastically lower the cost of implementing VOT. The per-patient cost was notably higher in Haiti because of higher hardware cost at the beginning of the project; there was a learning curve when evaluating whether tablets or phones were best suited for the intervention.

Asynchronous VOT offsets some recurrent costs associated with synchronous VOT and improves flexibility by allowing persons with TB to record medication ingestion within an agreed range of time, even if they did not have internet access at that moment (*31*). Similarly, the use of compressed video files can lower data-use costs. Less worker time is needed if the recordings are reviewed at higher playback speed or if computer-assisted recognition of pill swallowing is used (*32*; J.N. Sekandi, unpub. data, ). However, those adaptations may not be sufficient to make VOT easily accessible to TB programs in low- and lower middle-income countries. Our scenario analysis suggests that the initial capital investment would have to be covered by donor funds for this technology to become cost saving to local TB programs in these settings.

Global DAT initiatives for TB are addressing the infrastructure cost burden by improving market access, procurement mechanisms, and supply chains (*33*). Our study complements this work by carefully documenting capital and operating expenditures, allowing for better planning and decisions by TB treatment programs (*34*). Our cost estimates for 99DOTS are similar to those from other TB programs that have used this technology (*15*,*35*). A recent study from the United States estimated that VOT was less expensive than in-person DOT provided by healthcare staff (*29*) for both the healthcare system and for persons with TB and their families. The extent that DATs offload HCWs from in-person observation will reduce their net cost.

However, local factors also further shape overall costs, such as the cost of internet and SMS (short message service), the DAT and platform used, specific infrastructure used and patient population served, labor costs, varying nature of services, and treatment duration. Barriers beyond internet connectivity and infrastructure include restricted availability, accessibility, and affordability of some technologies for persons in resource-limited areas and, similarly, the availability and affordability of technical personnel needed to support the TB clinics (*26*,*36*). Hence, real-world cost, effectiveness, and implementation data from high TB-incidence, lower-income settings will remain paramount.

In conclusion, advances in the usability and acceptability of DATs, coupled with widespread internet access and mobile phone use, make them viable tools for person-centered adherence support. However, economic evaluations are limited to date. Our analysis suggests that 99DOTS may be affordable to TB programs in diverse settings, particularly if used at scale. VOT appears less affordable for lower-income countries, although costs for both technologies could be reduced if the same infrastructure and hardware could support more patients, and both technologies would be cost saving should their fixed cost be covered by international donor funds. Our work is a step toward future cost-effectiveness analysis of DATs as more clinical outcome data become available.

Appendix Additional information for study of costs of digital adherence technologies for tuberculosis treatment support.
