# Supplementary material for: Costs of Digital Adherence Technologies for Tuberculosis Treatment Support, 2018–2021
Source: Emerg Infect Dis. 2024 Jan;30(1):79–88. doi: 10.3201/eid3001.230427 (PMC10756355; doi:10.3201/eid3001.230427)
Supplement: Appendix — Additional information for study of costs of digital adherence technologies for tuberculosis treatment support. [file 23-0427-Techapp-s1.pdf]

Article DOI: https://doi.org/10.3201/eid3001.230427

EID cannot ensure accessibility for supplementary materials supplied by authors. Readers who have difficulty accessing supplementary content should contact the authors for assistance.

Costs of Digital Adherence Technologies for Tuberculosis Treatment Support, 2018–2021

Appendix

99DOTS tool and data

Appendix Table 1. Costing tool - 99DOTS

Cost components	Details/Formula *
Project and respondent information	
1. Name of the project	Text
2. Country	Text
3. Name of respondent	Text
4. Position of respondent	Text
5. e-mail of respondent	Text
6. Phone	Text
7. Local currency used in the country	Text
8. Date the questionnaire was filled	YYYY - MM - DD
9. Was anything donated to the project? (e.g., equipment, technical support, training sessions offered as donations, etc.)	Yes, No
10. List detailed items and services that were donated to the project	Text
Population	
I. Number of patients who used 99DOTS during the project	N
a) Phone and accessories	For patients
A1. Number of phones that were purchased	N
A2. Unit cost of phone purchased	$
A3. Number of SIM cards that were purchased	N
A4. Unit cost of SIM card	$
A5. Number of charger or solar batteries that were purchased	N
A6. Unit cost of charger or solar batteries	$
A7. Number of phone lines provided with airtime	N
A8. Total cost for airtime per phone line for the entire duration of the project	$
A9. Total cost of phones (includes phone, SIM card and airtime)	(A1*A2) + (A3*A4) + (A5*A6) + (A7*A8)
Per patient cost for phone and accessories	A9/I
b) Platform / infrastructure costs	
B1. Initial configuration/customization costs (setting up phonelines and or platform at sites)	$
B2. Type of configuration (portable, toll-free or SMS)	Text
B3. Total fixed cost of renting toll free phone lines / SMS line or any other system used to support 99DOTS platform for the entire duration of the project	$
Per patient fixed cost of the platform/infrastructure	(B1 + B3)/I
c) Medication sleeves	
C1. Total cost of 99DOTS medication sleeve printing (including secondary packaging, labels, etc.)	$
C2. Total cost for shipping of 99DOTS medication sleeves (including customs, etc.)	$
Per patient cost for medication sleeves	(C1+C2)/I
d) Medication Preparation	
D1. Typical amount of time to wrap and prepare one medication medication sleeve (in minutes)	Minutes
D2. Type of staff wrapping medication	Job Category
D3. Wage of staff (per hour)	$
D4. Cost of labor for wrapping per medication sleeve (Total time for wrapping an medication sleeve)	D1*D3/60

Cost components	Details/Formula *
D5. Number of medication sleeves required per patient for the entire duration of treatment	N
Per patient preparation cost for entire duration of treatment	D4*D5
e) 99DOTS Calls/SMS costs	
E1. Average number of calls/SMS made by a patient to 99DOTS during the entire duration of treatment	N
E2. Amount the project paid per call/SMS made by a patient to 99DOTS	$
E3. Per patient call/SMS to 99DOTS cost for entire duration of treatment	E1*E2
E4. Average number (per patient) of 99DOTS reminder/education SMS sent by the system for missed doses	N
E5. cost the project has to pay per 99DOTS system SMS	$
E6. per patient cost of 99DOTS adherence reminders for the entire duration of treatment	E4*E5
Total per patient cost (TO AND FROM) 99DOTS system call/SMS for entire duration of treatment	E3 + E6
f) Adherence monitoring by HCW using 99DOTS platform	
F1. Type of HCW who does adherence monitoring using 99DOTS platform	Job Category
F2. HCW wage (per hour)	$
F3. Typical amount of time (in min) spent by HCW monitoring adherence on the platform per patient (duration of project)	N
Per patient cost of 99DOTS adherence monitoring	F3*F2/60
g) Systems, Data management and technical support	
G1. Amount spent for staff working on data management for 99DOTS platform (salaries paid for support of DAT platform)	$
G2. Number of days that technical support was provided, or number of times specific technical services were provided (beyond initial setup)	N
G3. Cost per day or per activity reported in G2	$
G4. Total cost of technical support for 99DOTS platform	G2*G3
G5. Amount paid for health facility hardware (laptops, desktop, or tablet computer to track adherence)	$
G6. Payments made for monthly plans for technical services such as data plans and phone plans for HCW and health facility	$
Per patient cost for Systems, Data management and technical support	(G1 + G4 + G5 + G6)/I
h) Escalation in case of non-adherence and HCW response	(improve adherence)
Escalation related phone calls:	
H1. Number of patients who required follow up by phone as part of adherence escalation procedure	N
H2. Average number of phone calls made by HCW per patient (for entire duration of the project)	N
H3. Average HCW time per call (in minutes) required to speak to a patient who requires adherence follow up	minutes
H4. Total time (in minutes) spent by HCW with patients on a phone for adherence follow up	H1*H2*H3
H5. Type of HCW who usually makes phone calls	Job Category
H6. Average HCW wage (per hour)	$
H7. Additional fees associated with phone call follow up (per call)	$
H8. Total cost for Phone calls	(H4*H6/60min) + (H1*H2*H7)
Escalation related Home Visits:	
H9. Number of patients who required a home visit as part of escalation procedure	N
H10.Average number of home visits per individual who requires home visits	N
H11.Amount of time per visit (including travel)	minutes
H12. Total time (in minutes) spent by HCW on home visits for patients who required adherence follow up	H9*H10*H11
H13.Type of HCW doing home visit	Job Category
H14.HCW wage (per hour)	$
H15.Additional costs for HCW per visit (e.g., Travel/transport costs, incentives, etc.)	$
H16. Total cost for home visits	(H12*H14/60) + (H9*H10*H15)
Per patient cost for escalation activity	(H8 + H16)/I
i) DAT Training for HCWs *(Trainee costs)*	List up to three
1st Category of HCW trained	Job Category 1
I1.1 Number of Category 1 trained	N
I1.2 Typical amount of time for training (in minutes) (per HCW in category 1)	Minutes
I1.3 Total time in minutes for training all HCW in category 1	I1.1*I1.2
I1.4 HCW wage (per hour)	$
I1.5 Total cost for training all HCW in category 1	I1.3*I1.4/60
2nd Category of HCW trained	Job Category 2
I2.1 Number of Category 2 trained	N
I2.2 Typical amount of time for training (in minutes) (per HCW in category 2)	Minutes
I2.3 Total time in minutes for training all HCW in category 2	I2.1*I2.2
I2.4 HCW wage (per hour) (category 2)	$
I2.5 Total cost for training all HCW in category 2	I2.3*I2.4/60
3rd Category of HCW trained	Job Category 3
I3.1 Number of Category 3 trained	N
I3.2 Typical amount of time for training (in minutes) (per HCW in category 3)	Minutes

Cost components	Details/Formula *
I3.3 Total time in minutes for training all HCW in category 3	I3.1*I3.2
I3.4 HCW wage (per hour) (category 3)	$
I3.5 Total cost for training all HCW in category 3	I3.3*I3.4/60
I4. Total cost for training	I1.5 + I2.5 +I3.5
Per patient training cost	I4/III
j) Additional training costs *(Trainer costs)*	
Choose the training scenario for your project	
Scenario I: training was provided by individuals who were paid for each training session	
J1. Most common job category of staff providing the training sessions for HCW on how to use the 99DOTS	Job category (trainer)
J2. Typical amount of time spent by a trainer on training (including preparation and delivery) (in hours)	Hours
J3. Approximate hourly salary of someone doing training (trainer)	$
J4. Number of trainers	N
J5. Total trainers' wage directly related to 99DOTS training	J2*J3*J4
Scenario II: training was conducted by an outside organization or paid as a package	
J6. Total trainers' cost	$
J7. Additional expenditures related to training (including travel, training venue, subsistence for trainers and trainees, etc. but not time spent by, or salary paid to trainers or trainees)? Specify in column C and enter the cost in column C	$
Per patient cost of running training sessions (not including time for HCW who are being trained)	IF Scenario I: (J5 + J7)/III<br>IF Scenario II: (J6 + J7)/III

Total per patient cost for 99DOTS is the SUM of the following categories	Per patient cost for phone and accessories<br>Per patient fixed cost of the platform/infrastructure<br>Per patient cost for medication sleeves<br>Per patient preparation cost for entire duration of treatment<br>Total per patient cost (TO AND FROM) 99DOTS system call/SMS for entire duration of treatment<br>Per patient cost of 99DOTS adherence monitoring<br>Per patient cost for Systems, Data management and technical support<br>Per patient cost for escalation activity<br>Per patient training cost<br>Per patient cost of running training sessions (not including time for HCW who are being trained)

*Text in blue Indicates calculated fields. N = Numeric.

Appendix Table 2. Cost components for 99DOTS across the three study populations

Cost components	Bangladesh	The Philippines	Tanzania
Population			
I. Number of patients who used 99DOTS during the project	719	396	976
a) Phone and accessories			
A1. Number of phones that were purchased	22	0	0
A2. Unit cost of phone purchased	$354.58	$0	$0
A3. Number of SIM cards that were purchased	22	296	0
A4. Unit cost of SIM card	$11.79	$1.00	-
A5. Number of charger or solar batteries that were purchased	0	0	0
A6. Unit cost of charger or solar batteries	$0.00	-	-
A7. Number of phone lines provided with airtime	10	0	0
A8. Total cost for airtime per phone line for the entire duration of the project	$70.74	-	-
A9. Total cost of phones (includes phone, SIM card and airtime)	$8,767.63	$296.00	-
Per patient cost for phone and accessories	$12.19	$0.75	-
b) Platform / infrastructure costs			
B1. Initial configuration/customization costs (setting up phonelines and or platform at sites)	$ 10,304.5	$ 13,000.0	$8,736.3
B2. Type of configuration (portable, toll-free or SMS)	-	toll-free SMS	-
B3. Total fixed cost of renting toll free phone lines / SMS line or any other system used to support 99DOTS	$4,467.2	$901.3	$1,772.8
Per patient fixed cost of the platform/infrastructure	$20.54	$35.10	$10.77
c) Medication sleeves			
C1. Total cost of 99DOTS medication sleeve printing (including secondary packaging, labels, etc.)	$4,922.33	$13,000	$3,628
C2. Total cost for shipping of 99DOTS medication sleeves (including customs, etc.)	$70.74	$370.00	$6,120.00
Per patient cost for medication sleeves	$ 6.94	$33.76	$9.99

Cost components	Bangladesh	The Philippines	Tanzania
d) Medication Preparation			
D1. Typical amount of time to wrap and prepare one medication sleeve (in minutes)	2	1	1
D2. Type of staff wrapping medication	Research	TB nurse	Nurse
D3. Wage of staff (per hour)	$1.36	$1.59	$1.09
D4. Cost of labor for wrapping per medication sleeve (Total time for wrapping an medication sleeve)	$0.05	$0.03	$0.02
D5. Number of medication sleeves required per patient for the entire duration of treatment	35	24	24
Per patient preparation cost for entire duration of treatment	$1.58	$0.64	$0.44
e) 99DOTS Calls/SMS costs			
E1. Average number of calls/SMS made by a patient to 99DOTS during the entire duration of treatment	180	128	99
E2. Amount the project paid per call/SMS made by a patient to 99DOTS	$0.00	$0.01	$0.03
E3. Per patient call/SMS to 99DOTS cost for entire duration of treatment	-	$1.02	$3.27
E4. Average number (per patient) of 99DOTS reminder/education SMS sent by the system for missed doses	50	53	190
E5. cost the project has to pay per 99DOTS system SMS	$0.01	$0.01	$0.03
E6. per patient cost of 99DOTS adherence reminders for the entire duration of treatment	$0.59	$0.42	$5.70
Total per patient cost (TO AND FROM) 99DOTS system call/SMS for entire duration of treatment	$0.59	$1.45	$8.97
f) Adherence monitoring by HCW using 99DOTS platform			
F1. Type of HCW who does adherence monitoring using 99DOTS platform	Research	TB nurse	Nurse
F2. HCW wage (per hour)	$1.36	$1.59	$1.09
F3. Typical amount of time (in min) spent by HCW monitoring adherence on the platform per patient	1080	504	60
Per patient cost of 99DOTS adherence monitoring	$24.41	$13.36	$1.09
g) Systems, Data management and technical support			
G1. Amount spent for staff working on data management for 99DOTS platform (support of DAT platform)	$1,459.01	$1,639.21	$38,137.50
G2. Number of days that technical support was provided, or times specific technical services provided	35	15	6
G3. Cost per day or per activity reported in G2	$56.14	$74.50	$2,652.42
G4. Total cost of technical support for 99DOTS platform	$1,965.04	$1,117.50	$15,914.52
G5. Amount paid for health facility hardware (laptops, desktop, or tablet computer to track adherence)	$8,253.00	$449.00	$3,971.00
G6. Payments made for monthly plans for technical services such as data plans and phone plans for HCW	$589.50	$4.81	$143.48
Per patient cost for Systems, Data management and technical support	$17.06	$8.11	$59.60
h) Escalation in case of non-adherence and HCW response			
Escalation related phone calls:			
H1. Number of patients who required follow up by phone as part of adherence escalation procedure	17	31	736
H2. Average number of phone calls made by HCW per patient (for entire duration of the project)	10	1	9.92
H3. HCW time per call (in minutes) required to speak to a patient who requires adherence follow up	10	2	1
H4. Total time (in minutes) spent by HCW with patients on a phone for adherence follow up	1,700	62	7,304
H5. Type of HCW who usually makes phone calls	Research	TB nurse	Nurse
H6. Average HCW wage (per hour)	$1.36	$1.59	$1.09
H7. Additional fees associated with phone call follow up (per call)	$0.00	-	-
H8. Total cost for Phone calls	$38.42	$1.64	$132.69
Escalation related Home Visits:			
H9. Number of patients who required a home visit as part of escalation procedure	40	95	495
H10.Average number of home visits per individual who requires home visits	0	2	4
H11.Amount of time per visit (including travel)	180	45	120
H12. Total time (in minutes) spent by HCW on home visits for patients who required adherence follow up	-	8,550	237,600
H13.Type of HCW doing home visit	Research	Volunteer	Volunteers
H14.HCW wage (per hour)	$1.36	-	-
H15.Additional costs for HCW per visit (e.g., Travel/transport costs, incentives, etc.)	$1.77	$2.30	$1.79
H16. Total cost for home visits	-	$437.00	$3,544.20
Per patient cost for escalation activity	$0.05	$1.11	$3.77
i) DAT Training for HCWs *(Trainee costs)*			
1st Category of HCW trained	Research	TB nurse	Doctors
I1.1 Number of Category 1 trained	7	5	29
I1.2 Typical amount of time for training (in minutes) (per HCW in category 1)	1,440	300	600

Cost components	Bangladesh	The Philippines	Tanzania
I1.3 Total time in minutes for training all HCW in category 1	10,080	1,500	17,400
I1.4 HCW wage (per hour)	$1.36	$1.59	$2.40
I1.5 Total cost for training all HCW in category 1	$227.78	$39.75	$696.00
2nd Category of HCW trained	volunteer	Midwife	Clinicians
I2.1 Number of Category 2 trained	0	2	16
I2.2 Typical amount of time for training (in minutes) (per HCW in category 2)	0	300	600
I2.3 Total time in minutes for training all HCW in category 2	-	600	9,600
I2.4 HCW wage (per hour) (category 2)	-	$1.20	$1.09
I2.5 Total cost for training all HCW in category 2	-	$720.00	$10,464
Category of HCW trained	Research	TB Doctor	Nurses
I3.1 Number of Category 3 trained	1	3	56
I3.2 Typical amount of time for training (in minutes) (per HCW in category 3)	1,440	300	600
I3.3 Total time in minutes for training all HCW in category 3	1,440	900	33,600
I3.4 HCW wage (per hour) (category 3)	$3.12	$3.27	$1.09
I3.5 Total cost for training all HCW in category 3	$4,499.06	$2,943.00	$36,624
I4. Total cost for training	$4,726.85	$3,702.75	$47,784
Per patient training cost	$6.57	$9.35	$48.96
j) Additional training costs *(Trainer costs)*			
Choose the training scenario for your project	Scenario I	Scenario I& II	Scenario II
Scenario I: training was provided by individuals who were paid for each training session			
J1. Most common job category of staff providing the training sessions for HCW on how to use the 99DOTS	Research	IT	-
J2. Typical amount of time spent by a trainer on training (including preparation and delivery) (in hours)	40	240	-
J3. Approximate hourly salary of someone doing training (trainer)	$25.35	$6.00	-
J4. Number of trainers	6	3	-
J5. Total trainers' wage directly related to 99DOTS training	$6,083.64	$4,320.00	-
Scenario II: training was conducted by an outside organization or paid as a package			
J6. Total trainers' cost	-	$144.00	-
J7. Additional expenditures related to training (including travel, training venue, subsistence for trainers and trainees, etc. but not time spent by, or salary paid to trainers or trainees)? Specify in column C and enter the cost in column C	-	$788.00	$29,409.94
Per patient cost of running training sessions (not including time for HCW who are being trained)	$8.46	$15.25	$30.13
Total per patient cost for 99DOTS	$98.41	$118.88	$173.70

*Text in blue Indicates calculated fields - indicates non applicable components. N = Numeric.

Appendix Table 3. Total costs of 99DOTS during the implementation projects

Cost in USD	Bangladesh	The Philippines	Tanzania
Number of patients who used 99DOTS during the project	(n = 719)	(n = 396)	(n = 976)
Variable costs	$55,985	$33,173	$159,028
Phone and accessories	$8,768	$296	$0
Medication sleeves	$4,993	$13,370	$9,748
Medication preparation	$1,137	$252	$426
99DOTS Calls/SMS costs	$424	$573	$8,752
Adherence monitoring by HCW using 99DOTS platform	$17,547	$5,289	$1,064
Systems, Data management and technical support	$12,267	$3,211	$58,167
Escalation in case of non-adherence and HCW response	$38	$439	$3,677
DAT training for HCWs (Trainee costs)	$4,727	$3,703	$47,784
Additional training costs (Trainer costs)	$6,084	$6,040	$29,410
Fixed costs	$14,772	$13,901	$10,509
Platform / infrastructure costs	$14,772	$13,901	$10,509
Total overall cost of 99DOTS	$70,756	$47,074	$169,536

Appendix Table 4. Per person costs of 99DOTS during the implementation projects, without and with annuitization of capital costs§

	Project site			With costs annuitized over the 5-y life span of servers and phones		
Country	Bangladesh	The Philippines	Tanzania	Bangladesh	The Philippines	Tanzania
Number of patients who used 99DOTS during the project	(n = 719)	(n = 396)	(n = 976)	(n = 719)	(n = 396)	(n = 976)
Variable per patient cost	$78	$84	$163	$60	$83	$160
Per patient cost for phone and accessories *	$12	$0.75	$0.00	$3.51	$0.75	$0.00
Per patient cost for medication sleeves	$6.94	$34	$10	$6.94	$34	$10
Per patient medication preparation cost for entire duration of treatment	$1.58	$0.64	$0.44	$1.58	$0.64	$0.44
Total per patient cost of 99DOTS system call/SMS (TO AND FROM) for the entire duration of treatment	$0.59	$1.45	$8.97	$0.59	$1.45	$8.97
Per patient cost of 99DOTS adherence monitoring	$24	$13	$1.09	$24.4	$13	$1.09
Per patient cost for systems, Data management and technical support	$17	$8.11	$60	$7.88	$7.20	$56
Per patient cost for escalation activity	$0.05	$1.11	$3.77	$0.05	$1.11	$3.77
Per patient training cost for HCWs	$6.57	$9.35	$49	$6.57	$9.35	$49
Per patient cost of running training sessions (not including time for HCW who are being trained)	$8.46	$15	$30	$8.46	$15	$30
Fixed per patient cost*#	$21	$35	$11	$21	$8.84	$3.61
Total per patient cost of 99DOTS	$98	$119	$174	$81	$92	$163

§Totals from rounded totals.
*Equipment cost annuitized over a 5-y life span (phones, tablets and computers).
#Fixed cost consists of fixed cost of the platform/infrastructure.

VOT tool and data

Appendix Table 5. Costing tool - VOT

Cost components	Details/Formula *
Project and respondent information	
1. Name of the project``	Text
2. Country	Text
3. Name of respondent	Text
4. Position of respondent	Text
5. e-mail of respondent	Text
6. Phone	Text
7. Local currency used in the country	Text
8. Date the questionnaire was filled	YYYY - MM - DD
9. Was anything donated to the project? (e.g., equipment, technical support, training sessions offered as donations,etc.)	Yes, No
10. List detailed items and services that were donated to the project	Text
Population	
I. Type of patients (DS-TB or DR-TB)	Text
II. Length of a full course of treatment (in months)	N
III. Number of patients who were followed using DAT during the project	N
a) Phone and accessories	
A1. Number of phones that were purchased	N
A2. Unit cost of phone purchased	$
A3. Number of SIM cards that were purchased	N
A4. Unit cost of SIM card	$
A5. Number of charger or solar batteries that were purchased	N
A6. Unit cost of charger or solar batteries	$
A7. Number of phone lines provided with airtime	N
A8. Total cost for airtime per phone line for the entire duration of the project	$
A9. Total cost of phones (includes phone, SIM card and airtime)	(A1*A2) + (A3*A4) + (A5*A6) + (A7*A8)

Cost components	Details/Formula *
Per patient cost for phone and accessories	A9/III
b) Data	
B1. Average size of a video recording in Megabytes (MB)	N
B2. Cost per MB of data	$
B3. Total number of video calls/recordings made by all patients in the project	N
B4. Total cost for data for all patients	B1*B2*B3
Per patient cost for data	B4/III
c) Platform / infrastructure costs	
C1. Total amount spent on software license (if applicable) for the entire duration of the project	$
C2. Initial Configuration costs (setting up VDOT platform in country)	$
C3. Total amount spent to develop a VDOT software or platform for a project not using an already existing system	$
Per patient fixed cost of the platform/infrastructure	(C1 + C2 + C3)/III
d) Adherence monitoring by HCW using VDOT platform	
Routine adherence monitoring using VDOT	
D1. Type of HCW who does adherence monitoring using VDOT platform	Text
D2. HCW wage (per hour)	$
D3. Typical amount of time (in min) spent by HCW monitoring adherence on the platform per patient (duration of project)	N
D4. Per patient cost of VDOT routine adherence monitoring	D3*D2/60
Video recordings viewing	
D5. Type of HCW who usually views video recordings	Text
D6. HCW wage (per hour)	$
D7. Average number of minutes per patient per recording	N
D8. Average number of recordings made by a patient in the project	B3/III
D9. Per patient average amount of time (in minutes) of recordings over the course of treatment	D7*D8
D10. Per patient cost of viewing video recordings	D9*D6/60
Per patient cost of VDOT adherence monitoring (routine + viewing of recordings)	D4 + D10
e) Systems, Data management and technical support	
E1. Amount spent for staff working on data management for VDOT platform (salaries paid for support of DAT platform)	$
E2. Number of days that technical support was provided or number of times specific technical services were provided (beyond initial setup)	N
E3. Cost per day or per activity reported in E2	$
E4. Total cost of technical support for VDOT platform	E2*E3
E5. Amount paid for health facility hardware (laptops, desktop or tablet computer to track adherence)	$
E6. Payments made for monthly plans for technical services such as data plans and phone plans for HCW and health facility	$
Per patient cost for Systems, Data management and technical support	(E1 + E4 + E5 + E6)/III
f) Escalation in case of non-adherence and HCW response	
Escalation related phone calls:	
F1. Number of patients who required follow up by phone as part of adherence escalation procedure	N
F2. Average number of phone calls made by HCW per patient (for entire duration of the project)	N
F3. Average HCW time per call (in minutes) required to speak to a patient who requires adherence follow up	minutes
F4. Total time (in minutes) spent by HCW with patients on a phone for adherence follow up	F1*F2*F3
F5. Type of HCW who usually makes phone calls	Job Category
F6. Average HCW wage (per hour)	$
F7. Additional fees associated with phone call follow up (per call)	$
F8. Total cost for Phone calls	(F4*F6/60min) + (F1*F2*F7)
Escalation related Home Visits:	
F9. Number of patients who required a home visit as part of escalation procedure	N
F10.Average number of home visits per individual who requires home visits	N
F11.Amount of time per visit (including travel)	minutes
F12. Total time (in minutes) spent by HCW on home visits for patients who required adherence follow up	F9*F10*F11
F13.Type of HCW doing home visit	Job Category
F14.HCW wage (per hour)	$
F15.Additional costs for HCW per visit (e.g., Travel/transport costs, incentives, etc.)	$
F16. Total cost for Home visits	(F12*F14/60) + (F9*F10*F15)
Per patient cost for escalation activity	(F8 + F16)/III
g) DAT Training for HCWs (Trainee costs)	
1st Category of HCW trained	Job Category 1
G1.1 Number of HCW trained	N
G1.2 Typical amount of time for training (in minutes) (per HCW in category 1)	Minutes
G1.3 Total time in minutes for training all HCW in category 1	G1.1*G1.2

Cost components	Details/Formula *
G1.4 HCW wage (per hour)	$
G1.5 Total cost for training all HCW in category 1	G1.3*G1.4/60
2nd Category of HCW trained	Job Category 2
G2.1 Number of HCW trained	N
G2.2 Typical amount of time for training (in minutes) (per HCW in category 2)	Minutes
G2.3 Total time in minutes for training all HCW in category 2	G2.1*G2.2
G2.4 HCW wage (per hour) (category 2)	$
G2.5 Total cost for training all HCW in category 2	G2.3*G2.4/60
3rd Category of HCW trained	Job Category 3
G3.1 Number of HCW trained	N
G3.2 Typical amount of time for training (in minutes) (per HCW in category 3)	Minutes
G3.3 Total time in minutes for training all HCW in category 3	G3.1*G3.2
G3.4 HCW wage (per hour) (category 3)	$
G3.5 Total cost for training all HCW in category 3	G3.3*G3.4/60
G4. Total cost for training	G1.5 + G2.5 +G3.5
Per patient training cost	G4/III
h) Additional training costs (Trainer costs)	
Choose the training scenario for your project	
Scenario I: training was provided by individuals who were paid for each training session	
H1. Most common job category of staff providing the training sessions for HCW on how to use the DAT	Job category (trainer)
H2. Typical amount of time spent by a trainer on training (including preparation and delivery) (in hours)	Hours
H3. Approximate hourly salary of someone doing training (trainer)	$
H4. Number of trainers	N
H5. Total trainers' wage directly related to VDOT training	H2*H3*H4
Scenario II: training was conducted by an outside organization or paid as a package	
H6. Total trainers' cost	$
H7. Additional expenditures related to training (including travel, training venue, subsistence for trainers and trainees, etc. but not time spent by or salary paid to trainers or trainees)? Specify in column C and enter the cost in column C	$
Per patient cost of running training sessions (not including time for HCW who are being trained)	IF Scenario I: (H5 + H7)/III<br>IF Scenario II: (H6+H7)/III
Total per patient cost for VDOT is SUM of the following categories	Per patient cost for smart phone<br>Per patient cost for data<br>Per patient fixed cost of the platform/infrastructure<br>Per patient cost of VDOT adherence monitoring by HCW<br>Per patient cost for Systems, Data management and technical support<br>Per patient cost for escalation activity<br>Per patient cost for HCW training<br>Per patient trainers' cost

*Text in blue Indicates calculated fields. N = Numeric.

Appendix Table 6. Cost components for VOT across the four study populations

Cost components	Haiti	Moldova (All)	Moldova (DS-TB)`	Moldova (DR-TB)	the Philippines
Population					
I. Type of patients (DS-TB or DR-TB)	DS-TB	All	DS-TB	DR-TB	DR-TB
II. Length of a full course of treatment (in months)	6	6 and 9	6	9	9
III. Number of patients who were supported using VOT during the project	87	308	173	135	110
a) Phone and accessories					
A1. Number of phones that were purchased	40	200	92	108	131
A2. Unit cost of phone purchased	$69.0	$110.0	$110.0	$110.0	$97.7
A3. Number of SIM cards that were purchased	60	140	65	75	111
A4. Unit cost of SIM card	$2.0	-	-	-	$0.8
A5. Number of charger or solar batteries that were purchased	0	-	-	-	90
A6. Unit cost of charger or solar batteries	$0.0	-	-	-	$1.8
A7. Number of phone lines provided with airtime	60	384	177	207	54
A8. Total cost for airtime per phone line for the entire duration of the project	$118.0	$27.8	$27.8	$27.8	$47.8
A9. Total cost of phones (includes phone, SIM card and airtime)	$9,960.0	$32,663.7	$15,048.8	$17,614.9	$15,635.7

Cost components	Haiti	Moldova (All)	Moldova (DS-TB)`	Moldova (DR-TB)	the Philippines
Per patient cost for phone and accessories	$114.5	$106.05	$86.99	$130.48	$142.1
b) Data					
B1. Average size of a video recording in Megabytes (MB)	60	30	30	30	-
B2. Cost per MB of data	$0.0	$0.0	$0.0	$0.0	-
B3. Total number of video calls/recordings made by all patients in the project	12,194	43,027	19,823	23,204	29,700
B4. Total cost for data for all patients	$731.6	$1,837.3	$846.46	$990.80	$0.0
Per patient cost for data	$8.4	$5.97	$4.89	$7.34	$0.0
c) Platform / infrastructure costs					
C1. Total amount spent on software license (if applicable) for the entire duration of the project	$31,149.0	-	-	-	$27,326.7
C2. Initial Configuration costs (setting up VDOT platform in country)	-	-	-	-	-
C3. Total amount spent to develop a VDOT software or platform for a project not using an already existing system	-	$40,000.0	$18,428.8	$21,571.2	-
Per patient fixed cost of the platform/infrastructure	$358.0	$129.87	$106.52	$159.79	$248.4
d) Adherence monitoring by HCW using VDOT platform					
Routine adherence monitoring using VDOT					
D1. Type of HCW who does adherence monitoring using VDOT platform	Field staff	Assistant	Assistant	Assistant	Nurse
D2. HCW wage (per hour)	$2.7	$11.0	$11.0	$11.0	-
D3. Typical amount of time (in min) spent by HCW monitoring adherence on the platform per patient (duration of project)	701	540	360	540	-
D4. Per patient cost of VDOT routine adherence monitoring	$31.5	$80.46	$66.00	$99.00	$0.0
Video recordings viewing					
D5. Type of HCW who usually views video recordings	Officer	Assistant	Assistant	Assistant	Nurse
D6. HCW wage (per hour)	$1.7	$11.0	$11.0	$11.0	$2.9
D7. Average number of minutes per patient per recording	0.3	2	2	2	10
D8. Average number of recordings made by a patient in the project	140.2	139.70	114.59	171.88	270.0
D9. Per patient average amount of time (in minutes) of recordings over the course of treatment	42.0	279	229	171.88	2,700.0
D10. Per patient cost of viewing video recordings #	$1.2	-	-	-	$132.7
Per patient cost of VDOT adherence monitoring (routine + viewing of recordings)	$32.7	$80.46	$66.00	$99.00	$132.7
e) Systems, Data management and technical support					
E1. Amount spent for staff working on data management for VDOT platform (salaries paid for support of DAT platform)	$34,200.0	-	-	-	-
E2. Number of days that technical support was provided or number of times specific technical services were provided (beyond initial setup)	220	160	74	86	-
E3. Cost per day or per activity reported in E2	$55.0	$28.5	$28.5	$28.5	-
E4. Total cost of technical support for VDOT platform	$12,100.0	$4,560.0	$2,100.88	$2,459.12	$0.0
E5. Amount paid for health facility hardware (laptops, desktop or tablet computer to track adherence)	$7,500.0	$650.0	$299.47	$350.53	$2,257.2
E6. Payments made for monthly plans for technical services such as data plans and phone plans for HCW and health facility	$0.0	$1,920.0	$884.58	$1,035.42	-
Per patient cost for Systems, Data management and technical support	$618.4	$23.15	$18.99	$28.48	$20.5
f) Escalation in case of non-adherence and HCW response					
Escalation related phone calls:					
F1. Number of patients who required follow up by phone as part of adherence escalation procedure	-	32	15	17	42
F2. Average number of phone calls made by HCW per patient (for entire duration of the project)	-	21	21	21	50
F3. Average HCW time per call (in minutes) required to speak to a patient who requires adherence follow up	-	5	5	5	15
F4. Total time (in minutes) spent by HCW with patients on a phone for adherence follow up	-	3,360	1,548	1,812	31,500
F5. Type of HCW who usually makes phone calls		Assistant	Assistant	Assistant	Nurse
F6. Average HCW wage (per hour)	-	$11.00	$11.00	$11.00	$2.95
F7. Additional fees associated with phone call follow up (per call)	-	-	-	-	-
F8. Total cost for Phone calls	$0.0	$616.0	$284.8	$332.20	$1,549
Escalation related Home Visits:					
F9. Number of patients who required a home visit as part of escalation procedure	-	0	0	0	18

Cost components	Haiti	Moldova (All)	Moldova (DS-TB)`	Moldova (DR-TB)	the Philippines
F10.Average number of home visits per individual who requires home visits	-	0	0	0	6
F11.Amount of time per visit (including travel)	-	0	0	0	240
F12. Total time (in minutes) spent by HCW on home visits for patients who required adherence follow up	-	-	-	-	25,920
F13.Type of HCW doing home visit	-	Assistant	Assistant	Assistant	-
F14.HCW wage (per hour)		$11.0	$11.0	$11.0	$3.2
F15.Additional costs for HCW per visit (e.g., Travel/transport costs, incentives, etc.)					
F16. Total cost for Home visits	$0.0	$0.0	$0.0	$0.0	$1,385.1
Per patient cost for escalation activity	$8.6	$2.00	$1.64	$2.46	$26.7
g) DAT Training for HCWs (Trainee costs)					
1st Category of HCW trained		Doctors	Doctors	Doctors	Nurse
G1.1 Number of HCW trained	-	43	43	43	6
G1.2 Typical amount of time for training (in minutes) (per HCW in category 1)	-	300	300	300	120
G1.3 Total time in minutes for training all HCW in category 1	-	12,900	12,900	12,900	720
G1.4 HCW wage (per hour)	-	$15.0	$15.0	$15.0	$2.9
G1.5 Total cost for training all HCW in category 1	$0.0	$3,225	$3,225	$3,225	$35.4
2nd Category of HCW trained		Assistant	Assistant	Assistant	Staff
G2.1 Number of HCW trained	-	53	53	53	8
G2.2 Typical amount of time for training (in minutes) (per HCW in category 2)	-	420	420	420	240
G2.3 Total time in minutes for training all HCW in category 2	-	22,260	22,260	22,260	1,920
G2.4 HCW wage (per hour) (category 2)	-	$11.0	$11.0	$11.0	-
G2.5 Total cost for training all HCW in category 2	$0.0	$4,081	$4,081	$4,081	$0.0
3rd Category of HCW trained	-	N/A	N/A	N/A	Nurse
G3.1 Number of HCW trained	-	-	-	-	4
G3.2 Typical amount of time for training (in minutes) (per HCW in category 3)	-	-	-	-	240
G3.3 Total time in minutes for training all HCW in category 3	-	-	-	-	960
G3.4 HCW wage (per hour) (category 3)	-	-	-	-	$2.9
G3.5 Total cost for training all HCW in category 3	$0.0	$0.0	$0.0	$0.0	$2,832
G4. Total cost for training	$1,201	$7,306	$3,366	$3,940	$2,867
Per patient training cost	$13.8	$23.72	$19.46	$24.93	$26.1
h) Additional training costs (Trainer costs)					
Choose the training scenario for your project					
Scenario I: training was provided by individuals who were paid for each training session					
H1. Most common job category of staff providing the training sessions for HCW on how to use the DAT	-	-	-	-	Doctor
H2. Typical amount of time spent by a trainer on training (including preparation and delivery) (in hours)	-	-	-	-	8
H3. Approximate hourly salary of someone doing training (trainer)	-	-	-	-	$30.8
H4. Number of trainers	-	-	-	-	1
H5. Total trainers' wage directly related to VDOT training	$0.0	$0.0	$0.0	$0.0	$246.2
Scenario II: training was conducted by an outside organization or paid as a package					
H6. Total trainers' cost	-	-	-	-	-
H7. Additional expenditures related to training (including travel, training venue, subsistence for trainers and trainees, etc. but not time spent by or salary paid to trainers or trainees)? Specify in column C and enter the cost in column C	-	-	-	-	$7,034.62
Per patient cost of running training sessions (not including time for HCW who are being trained)	$0.0	$0.0	$0.0	$0.0	$64.0
Total per patient cost for VDOT is SUM of the following categories	$1,154.4	$371.22	$304.49	$452.48	$660.5

*Text in blue Indicates calculated field. N = Numerics. - indicates non applicable components

[#]D10 was intentionally removed from Moldova study because it would have double counted the per patient cost of VDOT adherence monitoring

Appendix Table 7. Total costs of VOT at project sites

Cost in USD	Haiti	Moldova DS-TB	Moldova DR-TB	Moldova (All TB)	The Philippines
Number of patients who used VOT during the project	(n = 87)	(n = 173)	(n = 135)	(n = 308)	(n = 110)
Variable cost	$69,287	$34,248	$40,088	$74,336	$45,330
Phone and accessories	$9,960	$15,049	$17,615	$32,664	$15,636
Data	$732	$846	$991	$1,837	$0
Adherence monitoring by HCW using VOT platform	$2,845	$11,418	$13,365	$24,783	$14,601
Systems, Data management and technical support	$53,800	$3,285	$3,845	$7,130	$2,257
Escalation in case of non-adherence and HCW response	$750	$284	$332	$616	$2,934
DAT Training for HCWs (Trainee costs)	$1,201	$3,366	$3,940	$7,306	$2,867
Additional training costs (Trainer costs)	$0	$0	$0	$0	$7,035
Fixed cost	$31,149	$18,429	$21,571	$40,000	$27,327
Platform / infrastructure	$31,149	$18,429	$21,571	$40,000	$27,327
Total overall cost of VOT	$100,436	$52,677	$61,659	$114,336	$72,656

Appendix Table 8. Per person VOT costs

	Project population				
Country	Haiti	Moldova DS-TB	Moldova DR-TB	Moldova (All TB)	The Philippines
Number of patients who used VOT during the project	(n = 87)	(n = 173)	(n = 135)	(n = 308)	(n = 110)
Variable per patient cost	$796	$198	$293	$242	$412
Per patient cost for phone and accessories*	$114	$87	$130	$106	$142
Per patient cost for data	$8.41	$4.89	$7.34	$5.97	$0.00
Per patient cost of VOT adherence monitoring	$33	$66	$99	$80	$133
Per patient cost for systems, Data management and technical support	$618	$19	$28	$23	$21
Per patient cost for escalation activity	$8.62	$1.64	$2.46	$2.46	$27
Per patient training cost	$14	$19	$25	$24	$26
Per patient cost of running training sessions (not including time for HCW who are being trained)	$0.00	$0.00	$0.00	$0.00	$64
Fixed per patient cost*#	$358	$107	$160	$130	$248
Total per patient cost of VOT	$1,154	$304	$452	$372	$661
	Project costs annuitized over the 5-y life span of servers and phones				
Country	Haiti	Moldova DS-TB	Moldova DR-TB	Moldova (All TB)	The Philippines
Number of patients who used VOT during the project	(n = 87)	(n = 173)	(n = 135)	(n = 308	(n = 110)
Variable per patient cost	$702	$151	$222	$185	$247
Per patient cost for phone and accessories*	$89	$40	$60	$49	$49
Per patient cost for data	$8.41	$4.89	$7.34	$5.97	$0.00
Per patient cost of VOT adherence monitoring	$33	$66	$99	$80	$133
Per patient cost for systems, Data management and technical support	$549	$19	$28	$23	$21
Per patient cost for escalation activity	$8.62	$1.64	$2.46	$2.46	$27
Per patient training cost	$14	$19	$25	$24	$5.2
Per patient cost of running training sessions (not including time for HCW who are being trained)	$0.00	$0.00	$0.00	$0.00	$13
Fixed per patient cost*#	$358	$21	$32	$26	$248
Total per patient cost of VOT	$1,060	$172	$254	$211	$495

*Equipment cost annuitized over a 5-y life span (phones, tablets and computers).
#Fixed cost consists of fixed cost of the platform/infrastructure, and the cost of systems, data management and technical support.

Effectiveness and standard of care (DOT) visit schedules

Appendix Table 9. Effectiveness

Country	DAT	Study description	Effectiveness of DAT arm	Reference
Bangladesh	99DOTS	Implementation study of 99DOTS in private sector TB screening and treatment centers established by icddr,b under its social enterprise model in Dhaka. Total TB incidence 375,000	Overall adherence: 96% of prescribed doses taken	(*1*)

Country	DAT	Study description	Effectiveness of DAT arm	Reference
The Philippines	99DOTS	The study was designed to assess 99DOTS use in the private sector in the Philippines, where data suggests 50% of patients in the country seek care.<br>Total TB incidence 741,000	Overall adherence: 94% of prescribed doses taken #	(*2*)
United Republic of Tanzania	99DOTS	The study was done in mining communities in Tanzania in four districts and four regions. The intervention involves (i) provision of medication in 99DOTS sleeves, (ii) delivery of reminders via SMS<br>to patients, (iii) dosing histories used for counselling and for differentiated care (more intensive patient management), and (iv) targeted educational messaging based on adherence and risk factors via SMS or IVR.<br>Total TB incidence 132,000	Measured concordance with urine testing 95% of doses reported using 99DOTS were confirmed by urine testing . This concordance was highest (98%) in patients who were in their first 2 mo of treatment. §	(*3*)
Haiti	VOT	A feasibility, acceptance, persistence, accuracy and sustainability study of VOT for prisoners in a low-income country<br>Total TB incidence 18,000	Median adherence of 85.7% of doses taken, but limited to the 65 patients who completed treatment	(*4*)
The Philippines	VOT	This pilot study aimed to determine feasibility and acceptability of VOT in a high-burden, resource constrained DR-TB clinic in the Philippines where smartphones penetration is moderate and growing.<br>Total TB incidence 741 000	Good adherence was defined as ingestion of >90% of prescribed doses.<br>Observed doses:<br>Males<br>VOT 86.2%<br>Status quo 80.7%<br>Females<br>VOT 86.8%<br>Status quo 81.4%	(*5*)
Republic of Moldova	VOT	A pilot study to scale up locally developed VOT technology/program.<br>Total TB incidence 2,600	Overall: 89% adherence to TB treatment *<br>DS-TB 92%<br>DR-TB 85%	(*6*)

#Adherence Using 99DOTS calls as a proxy for adherence resulted in a 94% sensitivity using a urine test for isoniazid metabolites (IsoScreen) as the reference standard.
§Similar urinalysis approach used in Tanzania to that in the Philippines to assess adherence.
DAT; Digital adherence technology. DR-TB; drug resistant TB. DS-TB; drug susceptible TB. Icddr,b; International Centre for Diarrheal Disease Research, Bangladesh. KNCV; Koninklijke Nederlandse Centrale Vereniging tot bestrijding der Tuberculose. TB; Tuberculosis. VOT; video-observed treatment
*Adherence was measured by the proportion of days that a person with TB was observed ingesting medication during the planned treatment period

Appendix Table 10. DOT schedules and duration in the study settings – from the costing tool

	99DOTS sites			VOT sites			
Schedule and duration	Bangladesh	The Philippines	Tanzania	Haiti	Moldova DS-TB	Moldova DR-TB	The Philippines
Crude estimate for DOT cost for the standard of care - derived from the costing tool							
Duration of TB treatment (in months)	6	6	6	6	6	9	9
Frequency of nurse visits (for clinical purposes)	3	6	16	6	6	9	9
Frequency of physician visits (for clinical purposes)	2	6	6	6	6	9	36
Proportion on DOT	100%	100%	100%	100%	100%	100%	50%
Number of days DOT	120	168	120	120	120	180	180
Location of DOT - during the study	Health facility	Health facility	Home based	Prison	Health facility	Health facility	Health facility or Home
Hourly wage of person offering DOT support	$0.83	$1.40	$0.00	$4.60	$11.20	$11.20	$2.88
Duration of DOT visit - in minutes	45	45	0	45	15	15	2

References

<eref>1. Sultana S. Digital monitoring of tuberculosis (TB) treatment adherence for differentiated care in Bangladesh, an implementation research project [cited 2023 Nov 22]. https://theunion.org/our-work/conferences/history-of-the-union-world-conference-on-lung-health/conference-abstract-books</eref>

<eref>2. Digital Adherence KNCV. TB REACH Wave 6: KNCV digital adherence (99DOTS) project in the Philippines[cited 2023 Nov 22]. https://www.digitaladherence.org/wp-content/uploads/2020/08/TB-REACH-Philippines-003.pdf</eref>

<eref>3. Efo E, Onjare B, Shilugu L, Levy J, editors. Acceptability, feasibility and accuracy of 99DOTS adherence technology in mining region of Tanzania [cited 2023 Nov 22]. https://ieeexplore.ieee.org/document/9576971</eref>

<eref>4. Dirks LK. Video directly observed therapy for TB treatment in Haitian correctional facilities [cited 2023 Nov 22]. https://etd.library.emory.edu/concern/etds/gt54kp211?locale=en</eref>

<eref>5. Casalme DJO, Marcelo DB, dela Cuesta DM, Tonquin M, Frias MVG, Gler MT. Feasibility and acceptability of video observed therapy among multi-drug resistant tuberculosis patients in Cavite, Philippines. 2022 [cited 2023 Nov 22]. https://doi.org/10.5588/ijtld.21.0632</eref>

<eref>6. Celan C, Bivol S, Vilc V, Alexandru S, Severin L. Roll-out of video-supported treatment in Moldova to promote people-centered model of care [cited 2023 Nov 22]. https://doi.org/10.1183/13993003.congress-2021.OA3950</eref>
